# Supplementary material for: SARS-CoV-2 infection- induced seroprevalence among children and associated risk factors during the pre- and omicron-dominant wave, from January 2021 through December 2022, Thailand: A longitudinal study
Source: PLoS One. 2023 Apr 27;18(4):e0279147. doi: 10.1371/journal.pone.0279147 (PMC10138857; doi:10.1371/journal.pone.0279147)
Supplement: S1 Table — Sera samples were categized according to the dates of blood collection into pre- and omicron dominant wave. (DOCX) [file pone.0279147.s001.docx]

**Supplementary Information**

**S1 Table.** Baseline characteristics and data summaries of vaccination status and infection history of final study participants recruited from January to December 2021 (pre-omicron wave) and January to December 2022 (omicron-dominant wave). Sera samples were categized according to the dates of blood collection into pre- and omicron dominant wave.

| **Characteristics** | **Pre-omicron wave**  **(n=241)** | **Omicron wave**  **(n=201)** |
| --- | --- | --- |
| Age, mean (S.D.) | 5.25 (0.4) | 6.2 (0.4) |
| Gender, n (%)  Girl  Boy | 123 (51.0%)  118 (49.0%) | 103 (51.2%)  98 (48.8%) |
| History of SARS-CoV-2 infection, n (%)  Previous SARS-CoV-2 infection  No previous infection  N/A | -  -  241(100%) | 66 (32.8%)  116 (57.7%)  19 (9.5%) |
| Vaccination status, n (%)  Unvaccinated  Vaccination  BNT162b2 (1 dose)  BNT162b2 (2 doses)  BNT162b2 (3 doses) | 241 (100%)  -  -  -  - | 109 (54.2%)  92 (45.8%)  37 (40.2%)  53 (57.6%)  2 (2.2%) |
| Household member, n (%)  2-4  ≥ 5  N/A | 99 (41.1%)  69 (28.6%)  73 (30.3%) | 99 (49.3%)  69 (34.3%)  33 (16.4%) |
| Confirmed COVID-19 infection in household members tested via PCR or ATK, n  No  Yes  N/A | -  -  241(100%) | 70 (34.8%)  97 (48.3%)  34 (16.9%) |

Previous SARS-CoV-2 infection was tested by PCR/ATK; N/A : No data available.
