# Supplementary material for: SARS-CoV-2 infection- induced seroprevalence among children and associated risk factors during the pre- and omicron-dominant wave, from January 2021 through December 2022, Thailand: A longitudinal study
Source: PLoS One. 2023 Apr 27;18(4):e0279147. doi: 10.1371/journal.pone.0279147 (PMC10138857; doi:10.1371/journal.pone.0279147)
Supplement: S2 Table — (DOCX) [file pone.0279147.s002.docx]

**Supplementary Information**

**S2 Table.** Number of SARS-CoV-2 infection-induced seropositive and seronegative per month detected in children aged 5-7 years old between pre- (January-December 2021) and omicron dominant wave (January-December 2022)

|  | Pre-omicron wave  (January-December 2021) | | | Omicron wave  (January-December 2022) | | |
| --- | --- | --- | --- | --- | --- | --- |
| months | Infection-induced seropositive ^a^ | Infection-induced seronegative | Total | Infection-induced seropositive ^a^ | Infection-induced seronegative | Total |
| January | 0 | 24 | 24 | 2 | 22 | 24 |
| February | 0 | 14 | 14 | 2 | 9 | 11 |
| March | 0 | 23 | 23 | 7 | 5 | 12 |
| April | 0 | 17 | 17 | 6 | 3 | 9 |
| May | 0 | 21 | 21 | 14 | 11 | 25 |
| June | 0 | 21 | 21 | 13 | 21 | 34 |
| July | 0 | 0 | 0 | 12 | 8 | 20 |
| August | 3 | 12 | 15 | 6 | 4 | 10 |
| September | 12 | 35 | 47 | 13 | 7 | 20 |
| October | 4 | 20 | 24 | 10 | 3 | 13 |
| November | 1 | 16 | 17 | 8 | 9 | 17 |
| December | 2 | 16 | 18 | 5 | 1 | 6 |
| **Total** | **22** | **219** | **241** | **98** | **103** | **201** |

^a^ Infection-induced seropositivity was estimated based on the presence of anti-RBD Ig (≥ 0.8 U/mL), anti-RBD IgG (≥7.1 BAU/mL) or anti-N IgG (≥ 1.4 S/C) among unvaccinated children and the presence of anti-N IgG in vaccinated individuals with BNT162b2 vaccine.
